# Breast Cancer Prognosis Prediction and Immune Pathway Molecular Analysis Based on Mitochondria-Related Genes

**DOI:** 10.1155/2022/2249909

**Published:** 2022-05-31

**Authors:** Weixu Luo, Yuanshan Han, Xin Li, Zhuo Liu, Pan Meng, Yuhong Wang

**Affiliations:** ^1^Institute of Innovation and Applied Research in Chinese Medicine, Hunan University of Chinese Medicine, Changsha 410208, China; ^2^Department of Pharmacy, The Third Hospital of Changsha, Changsha 410015, China; ^3^The First Affiliated Hospital, Hunan University of Chinese Medicine, Changsha 410007, China; ^4^Hunan Academy of Traditional Chinese Medicine Affiliated Hospital, Changsha 410006, China

## Abstract

**Background:**

Mitochondria play an important role in breast cancer (BRCA). We aimed to build a prognostic model based on mitochondria-related genes.

**Method:**

Univariate Cox regression analysis, random forest, and the LASSO method were performed in sequence on pretreated TCGA BRCA datasets to screen out genes from a Gene Set Enrichment Analysis, Gene Ontology: biological process gene set to build a prognosis risk score model. Survival analyses and ROC curves were performed to verify the model by using the GSE103091 dataset. The BRCA datasets were equally divided into high- and low-risk score groups. Comparisons between clinical features and immune infiltration related to different risk scores and gene mutation analysis and drug sensitivity prediction were performed for different groups.

**Result:**

Four genes, MRPL36, FEZ1, BMF, and AFG1L, were screened to construct our risk score model in which the higher the risk score, the poorer the prognosis. Univariate and multivariate analyses showed that the risk score was significantly associated with age, M stage, and N stage. The gene mutation probability in the high-risk score group was significantly higher than that in the low-risk score group. Patients with higher risk scores were more likely to die. Drug sensitivity prediction in different groups indicated that PF-562271 and AS601245 might be new inhibitors of BRCA.

**Conclusion:**

We developed a new workable risk score model based on mitochondria-related genes for BRCA prognosis and identified new targets and drugs for BRCA research.

## 1. Introduction

Breast cancer (BRCA) is one of the most common cancers worldwide; it is also a complex disease with different types and molecular characteristics [1]. According to the expression status of hormone receptors (estrogen and progesterone receptors) and HER2, BRCAs are mainly classified into four subtypes: luminal *A*, luminal *B*, HER2-enriched, and triple-negative [2]. Although there are many different subtypes of BRCAs, it has been reported that some of these subtypes might convert to other under specific conditions [3]. Because BRCA is a complex heterogeneous disease, the pathogenesis and clinical manifestations of different patients may differ. Diagnostic testing of patients with cancer has not yet been fully integrated into the clinical practice [4]. In cases where the subtype of BRCA cannot be accurately determined, identifying prognostic risk factors may be an effective method for diagnosis and treatment [5]. Therefore, in this study, we aimed to construct a new prognostic model for BRCA.

Mitochondria are among the most important organelles. Except for highly specialized mammalian mature red blood cells, most eukaryotic cells have their own mitochondria. Oxidative phosphorylation during aerobic respiration occurs in the mitochondria; this process is the main source of cellular energy [6]. Mitochondria play an important role in BRCA. For example, metabolic patterns in cancer change according to the different needs of various solid tumors [7]. Gathering mitochondria, by altering their subcellular localization, results in an increase production of ROS, which are toxic to cells, weakening the invasive ability of BRCA cells [8]. In addition, as a cofactor of the electron transport chain in mitochondrial oxidative phosphorylation, an increase in heme synthesis could inhibit glycolysis and oxidative metabolism, reducing the proliferation of BRCA cells [9]. Besides, many mitochondria-related genes play important roles in cancer. For example, BMF is a proapoptotic gene. In BRCA cell lines MCF-7 and MDA-MB-231, BMF could bind Bcl-2, instead of Bax, leading to mitochondrial outer membrane permeabilization and, finally, to apoptosis [10]. Based on the importance of mitochondria and related genes, we wondered whether we could construct a prognostic model for BRCA using mitochondria-related genes.

The mutation risk of moderate-penetrance genes in patients with BRCA is two to four times that in the general population [11]. Identifying high-risk germline mutations and implementing strategies to reduce risk could increase the survival rate of patients with BRCA [12]. Hence, we analyzed the mutation patterns of different risk score groups. It is difficult to screen useful drugs directly on patients with BRCA because of the high cost, great difficulty, and long periods of treatment needed. Fortunately, the differences between the expression patterns of tumor and normal cells can be used to predict the sensitivity of different cells to a certain drug. Additionally, it has been reported that pRRophetic is a workable tool for predicting drug effects in BRCA; therefore, we used these tools in our study [13]. There are various cell populations in the breast ductal epithelial layer and the normal breast tissue microenvironment, including immune and stromal cells. As their interactions play a major role in early BRCA [14], we performed immune infiltration analyses of patients with different risk scores.

In this study, we used TCGA BRCA datasets to screen four mitochondria-related genes, namely, MRPL36, FEZ1, BMF, and AFG1L, and constructed and verified a risk score model to predict the prognosis of BRCA. We also compared the features of the different risk score groups, such as mutation, drug sensitivity prediction, and immune infiltration.

## 2. Materials and Methods

### 2.1. Accessing and Preprocessing of BRCA Datasets

TCGA BRCA datasets were downloaded from the UCSC Xena platform (https://xenabrowser.net/), including RNA-seq data and clinical features of the patients, such as gender, age, stage, grade, survival status, and survival time. The external verification dataset GSE103091 (*n*=107) was downloaded from https://www.ncbi.nlm.nih.gov/geo/query/acc.cgi?acc=GSE103091. All probes in the microarray datasets were renamed and re-annotated. For probes with the same gene name, the mean value of the expression levels was defined as the gene's expression value. Data were normalized before performing any other analyses.

### 2.2. The Establishment of a Risk Score Model Based on Mitochondria-Related Genes

The mitochondria-related genes were collected from the Gene Set Enrichment Analysis, Gene Ontology: biological process (GSEA GO BP) gene set. Taking the survival time as one of the factors and the expression of the mitochondria-related genes as the other, univariate Cox analyses were carried out to calculate both the *p* value and hazard ratio. The *p*, *p* value indicated whether the gene was significantly related to the patients' survival time, whereas the hazard ratio indicated their relationships. A preliminary screening of the genes whose *p* value was >5 was performed, and further screening was done using random survival forest. After the error rate stabilized, we determined the importance of the genes in the upcoming prognostic model. After 1000 cross-validations using the LASSO method, the minimum lambda value was selected to determine how many genes would be used to establish the prognostic model. Finally, a group of genes was chosen based on their LASSO regression coefficients. The risk score consisted of the sum of the gene expression values multiplied by their LASSO regression coefficients.

(1) $$\begin{array}{c} \text{risk score}=\sum_{i=1}^{n}\left(\text{LASSO coefficient}\times \text{Gene expression}\right) \end{array}$$

### 2.3. Relationship between Clinical Features and Patient Risk Scores, and Mutation Analysis

All patient risk scores were calculated using the formula described above. Taking the risk score as one of the factors, univariate and multivariate Cox analyses were carried out with different clinical features, such as age or stage, as the other factor, to investigate the relationships between the risk score and each of these features. Patients in the BRCA datasets were equally divided into two groups according to their risk scores: low- and high-risk score groups. In addition, the mutation information from the patients was separately displayed by group to compare the mutation rates and types between them.

### 2.4. Immune Infiltration Analysis of Patients with Different Risk Scores

We performed four algorithms [15], ESTIMATE, McCounter, single-sample gene set enrichment analysis (ssGSEA), and TIMER, to estimate the relationship between the risk score and cell components or immune response. Heat maps were used to show differences in immune reactions under different algorithms.

### 2.5. Gene Set Enrichment Analysis (GSEA) and Drug Sensitivity Prediction

The gene sets used in this study were downloaded from the MSigDB (http://www.gsea-msigdb.org/gsea/msigdb). clusterProfiler (an R package) was used to perform the GSEA. pRRophetic (an R package) was used to predict the IC50, representing drug sensitivity [16], in the low- and high-risk score groups.

### 2.6. Statistical Analysis

All the statistical analyses in this study were performed using R 3.6.1 (https://www.r-project.org/). The Shapiro–Wilk test was used to check the normal distribution of variables. Unpaired Student's *t*-test was used to check the differences in variables that conformed to the normal distribution, whereas the Wilcoxon test was used to check the differences in variables that did not conform to the normal distribution. For multiple groups, one-way ANOVA was used to compare the mean values. The Kaplan–Meier method was used to generate survival curves using the R package “survminer.” All heat maps were generated using “pheatmap.”

## 3. Results

### 3.1. Establishment of a Risk Score Model to Predict the Prognosis of BRCA

**Figure 1:**
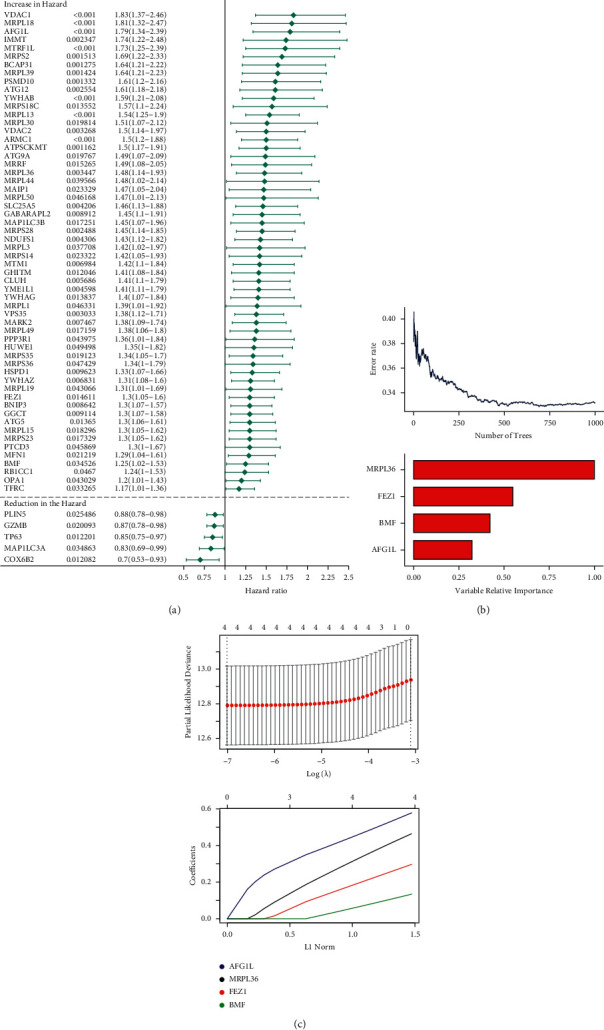
Screening of mitochondria-related genes and establishment of a risk score model. (a) Differential (increased or reduced) mitochondria-related gene expression in the hazard group compared with that in the normal group. (b) Error rates of randomly generated trees (upper panel). Variable relative importance of the four selected mitochondria-related genes. (c) LASSO analysis: partial likelihood deviance values were plotted against log (*λ*) (upper panel). The relative abundance of the selected genes varies with the risk score.

In the GSEA GO BP gene set, there were 345 genes related to mitochondria, 63 of which were screened out by univariate Cox analysis. The hazard ratios of these 63 genes are shown in Figure 1(a). Using the random forest algorithm (Figure 1(b)) and LASSO regression analysis (Figure 1(c)), we determined that the four genes with the highest variable relative importance were MRPL36, FEZ1, BMF, and AFG1L. Therefore, the LASSO coefficient of these four genes was used as the weight of the risk score model. The risk score of the prognostic model was built using the following formula:

(2) $$\begin{array}{c} \text{risk score}=0.464\times \text{MRPL}36+0.297\times \text{FEZ}1+0.134\times \text{BMF}+0.578\times \text{AFG}1\text{L} \end{array}$$

### 3.2. Prognosis Prediction of Different Risk Scores

**Figure 2:**
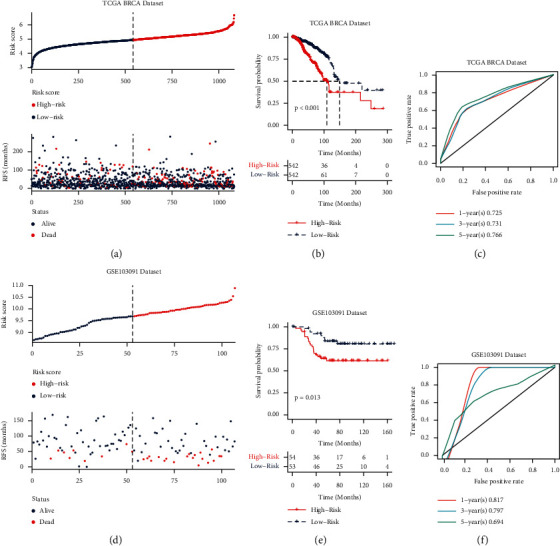
Establishment and verification of the risk score model. (a, d) Distribution of risk score and survival status in the corresponding datasets. (b, e) Survival analysis between high- and low-risk score groups in the different datasets. (c, f) 1-, 3-, and 5-year ROC curves for the different datasets. (a–c) The results from the analysis of the TCGA BRCA dataset (TCGA dataset); (d–f) results from the analysis of the GSE103091 dataset. RFS: relapse-free survival.

To verify whether the risk score model worked, we evaluated the prognosis of different risk scores in the BRCA and GSE103091 datasets. We found that the increase in the risk score was proportional to the increase in patient mortality (Figures 2(a), 2(d). In addition, the survival curves shown in Figures 2(b) and 2(e) show that the higher the risk score, the worse the prognosis of the patients (*P* < 0.05). Moreover, the ROC curves in Figures 2(c) and 2(f) show that the AUC values for one and three years were higher than 0.75, indicating that the risk score model was workable.

### 3.3. Relationships between Risk Scores and Clinical Characteristics, and Mutations in Different Risk Score Groups

**Figure 3:**
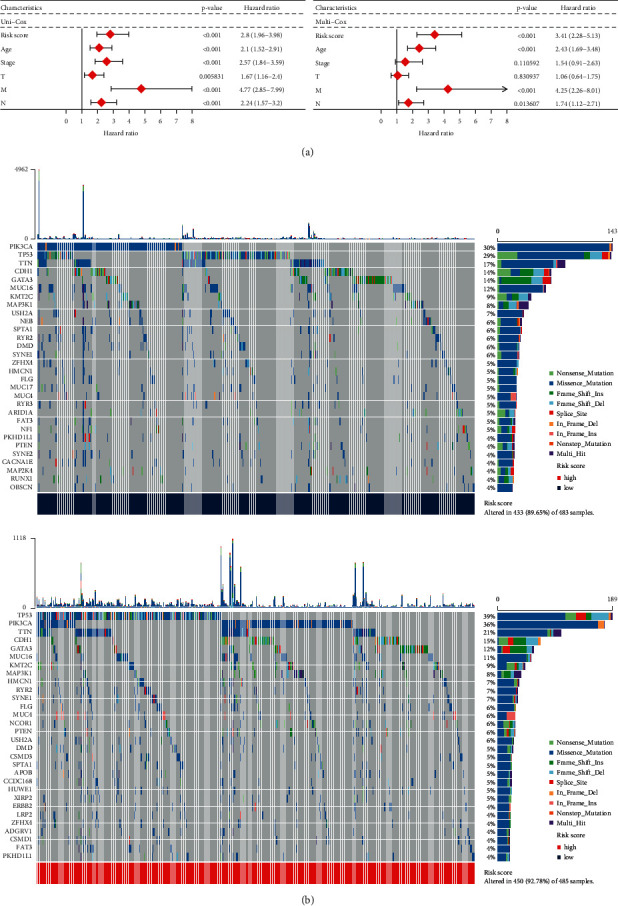
Univariate, multivariate, and mutation analyses of the different risk score groups. (a) Univariate and multivariate Cox analyses of the risk scores and the clinical characteristics of the patients. (b) Mutation patterns of mitochondria-related genes from different risk score groups.

The results from univariate and multivariate analyses of risk scores with clinical characteristics of the subjects included in TCGA BRCA datasets are shown in Figure 3(a). The univariate analysis showed that the risk score was significantly associated with age, stage, T stage, M stage, and N stage (*P* < 0.01). In addition, the multivariate analysis showed that the risk score was significantly related to age, M stage, and N stage (*P* < 0.01). All hazard ratios were greater than 1.

We performed mutation analysis of the low- and high-risk score groups, and the results were sorted by mutation patterns (Figure 3(b)). Comparing the upper and lower panels of Figure 3(b), it was found that patients in the high-risk score group had more mutations than those in the low-risk score group. Comparing the gene name on the left and the mutation rate on the right of Figure 3(b), the top-ranked gene mutations in the high- and low-risk score groups were roughly the same, in which the mutation probability of TP53, PIK3CA, TTN, CDH1, and GATA3 exceeded 10%. Remarkably, the mutation probability of HMCN1 increased from 5% in the low-risk score group to 7% in the high-risk score group. Comparing only the information on the right side of the panels in Figure 3(b), the pattern of mutations changed in the different groups. In the high-risk score group, there were more nonstop mutations in PIK3CA, fewer nonsense mutations in TP53, and more splice site and frameshift deletions in TP53 than in the low-risk score group. In addition, compared to the low-risk score group, fewer missense mutations and multihit mutations in CDH1, fewer frameshift insertions in GTAT3, fewer frameshift insertions and deletions in HMCN1, and more nonsense mutations in HMCN1 occurred in the high-risk score group. In particular, the in-frame insertion mutations in MUC4 were more abundantly found in the high-risk score group than in the low-risk score group. Thus, the mutation patterns differed between the high- and low-risk score groups.

### 3.4. Relationships between Risk Scores and Clinical Features, and Drug Sensitivity Prediction

**Figure 4:**
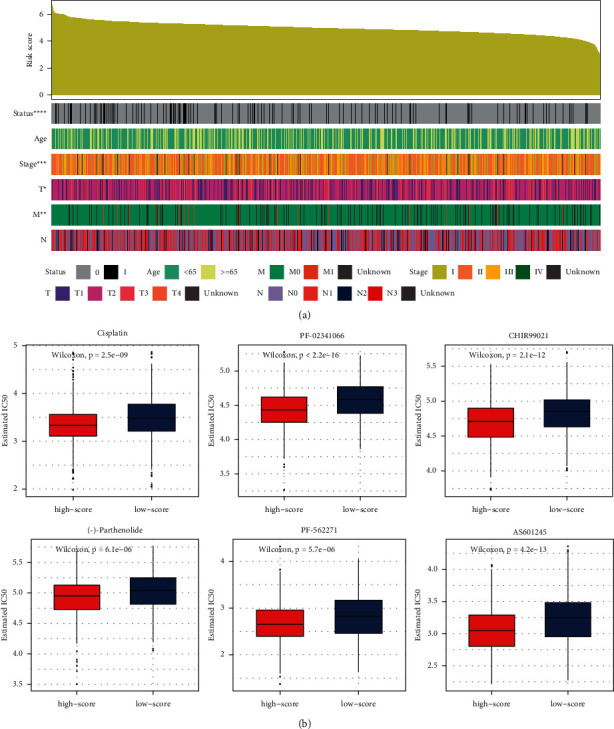
Clinical features and drug sensitivity prediction in breast cancer (BRCA) datasets. (a) Clinical characteristics of patients with BRCA, sorted by risk scores. (b) Predicted responses to drugs that might be used to treat BRCA.

As shown in Figure 4(a), as the risk scores increased, the death rate of the patients also increased significantly. In addition, an increase in the risk score corresponded to significant increases in the rate of stage IV and stage M1 BRCAs and to a significant decreased in stage T1 BRCA. The risk score of the patients was not related to age or N stage.

To identify potentially effective drugs against BRCA, we used pRRophetic to predict the different clinical drug responses in the high- or low-risk score groups (Figure 4(b)). Compared with the low-risk score group, the high-risk score group seemed more sensitive to cisplatin, crizotinib (PF-02341066), CHIR99021, (-)-parthenolide, AS601245, and PF-562271, indicating that these drugs could be used to treat BRCA.

### 3.5. Relationship between Risk Scores and Immune Infiltration

**Figure 5:**
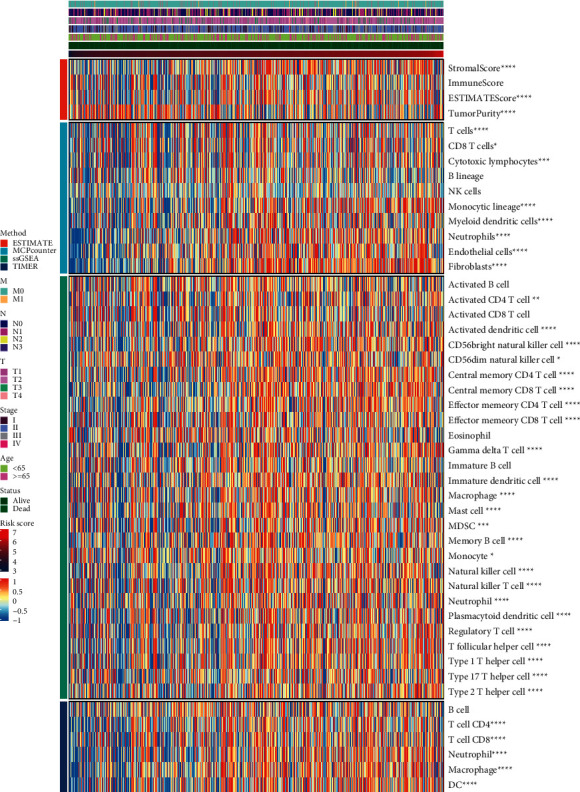
Immune infiltration analyses of BRCA datasets with different algorithms.

To analyze the differences in immune infiltration among patients with different risk scores, we used four methods: ESTIMATE, McCounter, single-sample gene set enrichment analysis (ssGSEA), and the TIMER algorithm (Figure 5). The epidemic immunity in BRCA was analyzed according to different gene expression patterns. According to the ESTIMATE analysis, tumor purity was negatively correlated with the risk score. In other words, the degree of immune cell infiltration increased at higher risk score values. Stromal and immune ESTIMATE scores significantly increased with increasing risk scores. According to the McCounter analysis, with an increase in risk scores, T cells, CD8 T cells, cytotoxic lymphocytes, monocytic lineage, myeloid dendritic cells, neutrophils, endothelial cells, and fibroblasts significantly increase as well. According to the ssGSEA analysis, with an increase in risk score, activated CD4 T cells, activated dendritic cells, CD 56 bright natural killer cells, CD56 dim natural killer cells, central memory CD4 T cells, central memory CD8 T cells, effector memory CD4 T cells, effector memory CD8 T cells, gamma delta T cells, immature dendritic cells, macrophages, mast cells, myeloid-derived suppressor cells, memory B cells, monocytes, natural killer cells, natural killer T cells, neutrophils, plasmacytoid dendritic cells, regulatory T cells, T follicular helper cells, type 1 T helper cells, type 17 T helper cells, and type 2 T helper cells were significantly increased. According to the TIMER analysis, with an increase in the risk score, CD4 T cells, CD8 T cells, neutrophils, macrophages, and dendritic cells were significantly more abundant. These results were consistent and showed that the risk scores were related to immune infiltration.

### 3.6. GSEA

**Figure 6:**
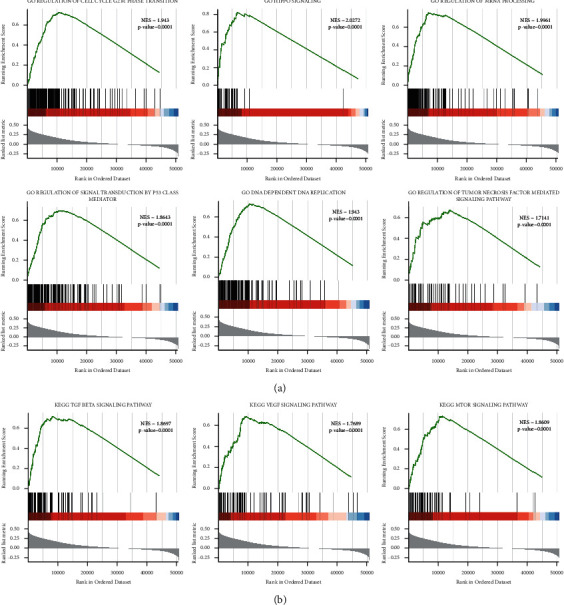
GSEA of the risk score model. (a) GSEA of GO processes. (b) GSEA of KEGG pathways. NES : normalized enrichment score.

We then chose several GO processes and KEGG pathways and performed GSEA. The results showed that the NES values of DNA-dependent DNA replication, the HIPPO signaling pathway, regulation of mRNA processing, the TNF-mediated signaling pathway, cell cycle G2 M phase transition, signal transduction by p53 class mediator (Figure 6(a)), the mTOR signaling pathway, the TGF*β* signaling pathway, and the VEGF signaling pathway (Figure 6(b)) were greater than 0 (*P* < 0.05), indicating that these processes or pathways were activated in the high-risk score group.

## 4. Discussion

In recent years, the combination of the random survival forest model and the LASSO regression method has been widely used to establish various disease prognostic models from different perspectives. Not only the gene expression patterns, imaging data, clinical characteristics [17], radiomics results [18], or other variables can also be used to establish prognostic models. Mitochondria play an important role in different cancers; mitochondria-related genes had also been used to build prognostic models of other diseases, for example, acute myeloid leukemia [19]. But this thinking perspective seems did not attract researchers' attention in previous breast cancer studies. We filled this research gap by constructing a prognosis risk score model based on four mitochondria-related genes. Besides its role in BRCA, BMF is also an important prognostic marker for patients with other cancers, such as colon cancer [20] and hepatocellular carcinoma [21]. MRPL36 is a mitochondrial ribosomal protein that has not been studied in BRCA, but MRPL36 has been correlated with poor progression-free survival in ovarian cancer [22]. The translation product of AFG1L, LACE1, interacts with p53 and mediates its mitochondrial translocation and apoptosis [23]. FEZ1 is an anti-invasive factor [24], and it is related to mitochondrial anterograde movement [25]. There is no research on FEZ1 in BRCA, but it is known that changes in the mitochondrial subcellular position in cells could affect the invasion ability of BRCA [8]. These four genes may be new useful targets for BRCA treatment or prognosis.

It is known that, after accumulating a certain number of mutations, somatic cells will develop into cancer cells that will be out of control and proliferate abnormally without limitation. In this study, we analyzed and compared the mutation patterns of the top 30 genes in the high- and low-risk score groups. TP53 is a well-known tumor suppressor gene, and its translation product, p53, is associated with apoptosis in cancer [26]. The loss of p53 leads to systemic neutrophil inflammation, which promotes BRCA metastasis [27]. PIK3CA is an important molecule in the PI3K signaling pathway, and its translation product, p110*α*, is a catalytic subunit of the PI3K*α* complex [2]. A PIK3CA-activating mutation often occurs in BRCA and causes disorders in the PI3K/Akt pathway. Therefore, PI3K inhibitors have been used to target PIK3CA for the treatment of different subtypes of advanced BRCA [28]. As the translation product of CDH1, E-cadherin is a structural molecule for cell adhesion and an important factor in maintaining epithelial characteristics. The regulation of cell migration is related to changes in E-cadherin subcellular localization [29, 30]. In some genetic contexts, AKT inhibitors may cause the accumulation of mutations in CDH1, thereby accelerating the metastasis and progression of BRCA [29]. Additionally, GATA3 is a transcription factor with a zinc-finger structure that regulates T-cell receptors in the immune system [31]. GATA3 is one of the most frequently mutated genes in BRCA; it can affect chromatin localization of FOXA1 and ER-*α* [32]. In BRCA, splice, frameshift, truncation, and extension are the most common mutations found in GATA3 [31]. The main function of GATA3 in the breast is to alter the fate of luminal cells [33]. The translation product of MUC4 is Mucin-4, a highly glycosylated protein, located on the surface of the cell membrane, that normally lubricates and protects vascular and epithelial surfaces [34]. It also promotes the combination of blood and tumor cells [35]. Therefore, Mucin-4 plays an important role in tumor progression and metastasis. In addition, an increased expression of Mucin-4 masks the HER2 epitope on the cell surface, resulting in trastuzumab resistance of cancer cells in patients with HER2-overexpressed BRCA [36]. Little research has focused on HMCN1 in breast cancer, but it is known that this gene is related to tumor heterogeneity and poor prognosis of BRCA [37]. The different mutations analyzed in our risk score model were consistent with those reported in other studies.

The assessment of tumor-infiltrating lymphocytes has been proven to be a reliable surrogate indicator and a powerful independent prognostic biomarker for immune antitumor activity in patients with BRCA [38]. Noteworthy, lung metastasis is a major cause of death in patients with BRCA. Neutrophil extracellular traps in the tumor microenvironment can promote this metastasis [39]. In BRCA, activated M2 macrophages can produce cytokines and promote tumor cell proliferation, metastasis, survival, and tumor angiogenesis [40]. Some cancer-associated fibroblast subpopulations can alter the immune microenvironment and lead to BRCA cell metastasis [41]. In some NRF2-positive breast cancers, immune cells, such as CD8+ T, CD4+ T, dendritic, and stromal cells (such as adipocytes, fibroblasts, and keratinocytes), are highly infiltrated [42]. In this study, we used several algorithms to evaluate the degree of immune infiltration. We found that, as the risk score increased, the infiltration of neutrophils and macrophages also increased. The results of the different algorithms in our study reached a consistent conclusion and coincided with those of previous reports.

Cisplatin is one of the most commonly used chemotherapeutic agents. It can block BRCA progression by blocking early epithelial-mesenchymal transition [43], preventing cancer cell division, and inducing apoptosis [44] or other mechanisms. Crizotinib (PF-02341066) is an FDA-approved small-molecule protein kinase inhibitor [45]. It can be used to treat BRCA by targeting the inhibition of MET and many other kinases [46]. CHIR99021 is an inhibitor of GSK3. Studies have shown that CHIR99021 inhibits the growth of the human BRCA cell line MDA-MB-231 [47]. (-)-Parthenolide is a sesquiterpene lactone isolated from Chrysanthemum morifolium. It can target FAK1 and affect the proliferation, survival, and movement of BRCA cells [48]. PF-562271 is a FAK inhibitor. Although it has not been reported in BRCA, it can inhibit the viability and migration ability of embryonal rhabdomyosarcoma cells [49]. Activation of JNK signaling in BRCA promotes the survival of cancer cells, and AS601245 is an inhibitor of JNK [50]. The screening done for all these drugs, according to the different risk score groups, is consistent with the results from other studies and suggests that PF-562271 and AS601245 may be used as new drugs for the treatment of BRCA.

Different biological processes and signaling pathways are involved in BRCA. For example, cancer stem-like cells in BRCA under DNA replication stress induce drug resistance and recurrence [51]. mRNA processing is usually disordered in BRCA [52]. In addition, the HIPPO signaling pathway plays an important role in BRCA metastasis through crosstalk with other signaling pathways [53]. Moreover, TNF-*α* is an important component of the tumor microenvironment of patients with BRCA; it is a proinflammatory cytokine secreted by macrophages or tumor cells. It can induce macrophages to differentiate into the M1 type, which is mainly used to kill tumors and promote chronic inflammation. TNF-*α* in peripheral blood has different implications for prognosis, depending on different expression patterns, together with other cytokines. The effect of TNF-*α* on BRCA cell proliferation or apoptosis depends on whether other pathways such as NF-*κ*B are active [54]. mTOR, downstream of the PI3K/AKT pathway, consists of two complexes with similar structures but different functions: mTORC1 and mTORC2. mTORC1 enhances inner cellular anabolism to promote cell growth, while mTORC2 participates in AKT phosphorylation. Inhibitors of mTORC1 or mTORC2, for example, everolimus, have been proven effective in treating the ER+/HER2+ subtype of BRCA [2]. Additionally, TGF*β* is a cytokine that promotes tissue repair and inhibits adaptive immunity, and plays an important role in epithelial-mesenchymal transition and tumor immune evasion [55]. The blockade of TGF*β* signaling in CD4+ T cells could restore the responses of helper T cells and inhibit cancer progression, which is mainly mediated by the induction of vascular system reorganization [56]. VEGF not only limits antitumor immunity but also promotes pathological angiogenesis in internal tumor tissues [57]. The above-mentioned studies are consistent with our predictions based on patients with different risk scores, according to our prognostic model.

Our prognostic model found that, in the high-risk score group, mutations, such as PI3KCA and TP53, often occur together with the activation of signaling pathways, such as the Hippo pathway or the PI3K/AKT/mTOR pathway. The wild-type p53 protein cooperates with the Hippo pathway to inhibit tumor cell growth by promoting senescence or apoptosis. This function could be reversed by the mutation status of the TP53 gene (which causes the p53 protein to gain GOF activity) [58]. The hotspot mutation H1047R in PI3KCA, frequently detected in BRCA, activates the PI3K/AKT/mTOR pathway and promotes BRCA tumor formation [59]. The described relationships between mutations and activated pathways are consistent with our results. Moreover, our risk score prognostic model may provide new insights into the specific mechanisms of other mutations in BRCA.

According to our model, patients with high-risk scores are sensitive to drugs such as cisplatin. Similarly, immune cells such as macrophages in patients with high-risk scores are affected. Some studies have shown that regulation of the immune microenvironment can enhance the immunological benefits of chemotherapy drugs such as cisplatin [60] and that cisplatin and other drugs can also regulate the proportion of immune cells in BRCA [61]. Interestingly, macrophage blockade enhances cisplatin response by releasing intratumoral type I interferons [62]. The described relationships between drugs and immune cells are consistent with our results. In addition, our risk score prognostic model may provide possible ideas for mechanistic studies of other chemotherapy drugs for BRCA.

Our study has some limitations. The same gene may have different prognostic significances in different BRCA subtypes. For example, mutations in TP53 may contribute to the mortality rate of luminal B or HER2-enriched subtype BRCAs but make no effort to treat luminal A subtype BRCA [63]. In the future, we intend to validate our model using different subtypes of BRCA datasets and explore the different expressions between patients with different risk scores in different datasets. This may provide new ideas and targets for BRCA treatment and prognosis of different subtypes.

## 5. Conclusions

In this study, we constructed a risk score model for BRCA prognosis based on four mitochondria-related genes. This model was evaluated from different perspectives, such as mutation, invasion, and sensitivity to drugs prediction, providing new insights for BRCA prognosis.

## Data Availability

TCGA Breast Cancer (BRCA) datasets (https://xenabrowser.net/) and GSE103091 were used to support this study.
